# Perturbation-based estimation of within-stride cycle metabolic cost

**DOI:** 10.1186/s12984-024-01424-8

**Published:** 2024-08-01

**Authors:** Alex C. Dzewaltowski, Prokopios Antonellis, Arash Mohammadzadeh Gonabadi, Seungmoon Song, Philippe Malcolm

**Affiliations:** 1 Department of Biomechanics and Center for Research in Human Movement Variability, University of Nebraska at Omaha, Omaha, NE USA; 2 Oregon Health & Science University, Portland, OR USA; 3 Rehabilitation Engineering Center, Institute for Rehabilitation Science and Engineering, Madonna Rehabilitation Hospital, Lincoln, NE USA; 4 Department of Mechanical and Industrial Engineering, Northeastern University, Boston, MA USA

## Abstract

Metabolic cost greatly impacts trade-offs within a variety of human movements. Standard respiratory measurements only obtain the mean cost of a movement cycle, preventing understanding of the contributions of different phases in, for example, walking. We present a method that estimates the within-stride cost of walking by leveraging measurements under different force perturbations. The method reproduces time series with greater consistency (*r* = 0.55 and 0.80 in two datasets) than previous model-based estimations (*r* = 0.29). This perturbation-based method reveals how the cost of push-off (10%) is much smaller than would be expected from positive mechanical work (~ 70%). This work elucidates the costliest phases during walking, offering new targets for assistive devices and rehabilitation strategies.

**Supplementary Information:**

The online version contains supplementary material available at 10.1186/s12984-024-01424-8.

## Introduction

**Fig. 1:**
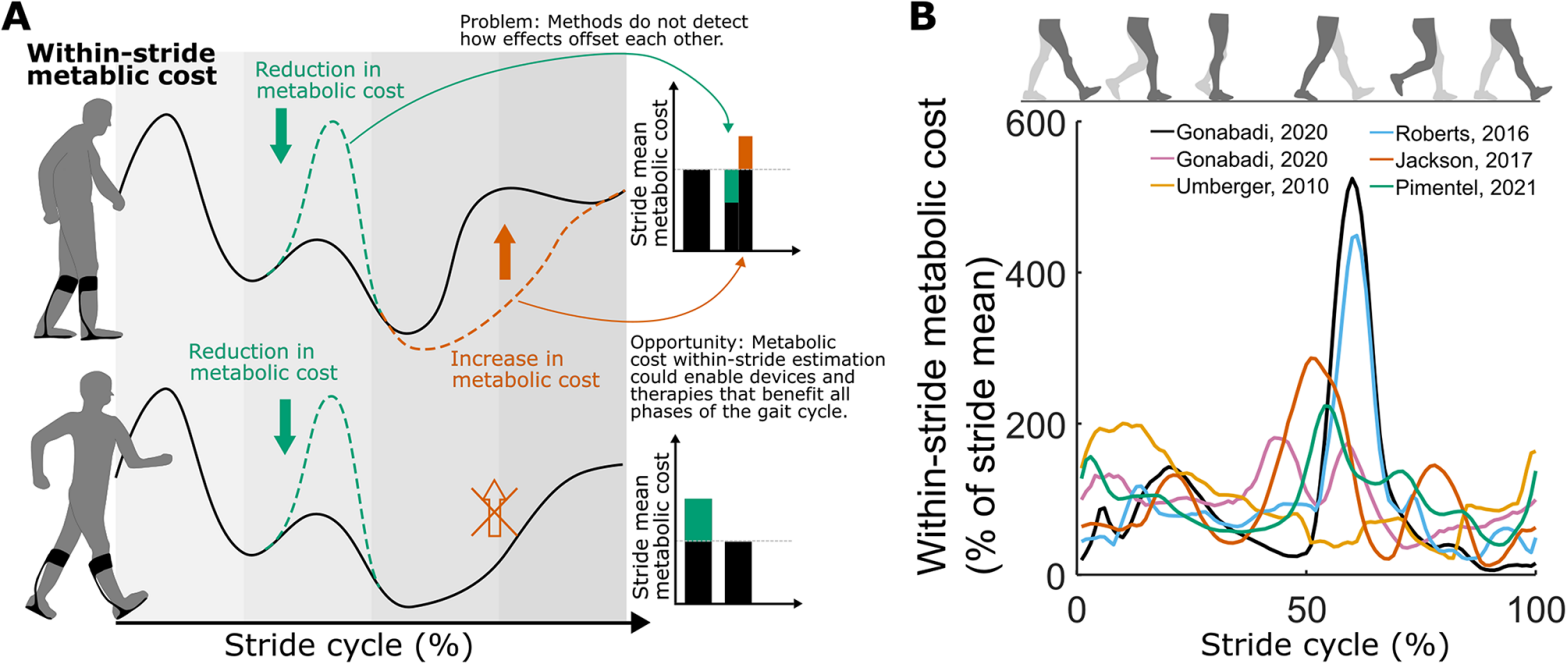
Motivation. (**A**). Limitation of assessing stride-mean metabolic cost using breath-by-breath measurements. The upper figure illustrates an intervention resulting in a cost reduction (depicted in green) during push-off and a cost increase (depicted in brown) during swing. The stride-mean metabolic cost (displayed in bars) does not enable differentiation of these effects. The lower section of the figure illustrates how comprehending the costs associated with various phases could facilitate the enhancement of interventions. (**B**). Limited consistency between estimations of within-stride metabolic cost using model-based methods. The mean correlation between estimations is 0.29 (95% confidence interval (CI) = 0.03–0.43) [16, 19, 38, 41, 42]

Metabolic cost is a critical measure used to characterize movement behavior [1–3]. Healthy walkers naturally adopt an energetically optimal stride cycle, for example, by walking with a step length [4] and knee flexion angle [5] that minimizes metabolic cost. Pathologies like stroke and cerebral palsy alter patients’ walking stride resulting in increases to metabolic cost by 60 to 300% [6, 7]. Such increases in metabolic cost correlate to drastic reductions in people’s mobility and overall quality of life [8, 9]. If we understand how stride cycle phases contribute to metabolic cost, therapies and devices may be better optimized to improve mobility (Fig. 1A).

Measurements of metabolic cost are too slow to detect the contributions of different stride phases. Current methods to calculate energy from oxidative reactions include measuring respiratory CO_2_ production by ingesting water with a radioisotope (‘doubly labelled water method’), measuring oxidative heat production using a chamber (‘direct calorimetry’), and measuring O_2_ consumption from respiration (‘indirect calorimetry’) [10]. Indirect calorimetry is the fastest and most commonly used method for measuring metabolic cost during locomotion; however, it still requires averaging several minutes of breaths to be reliable [11–13]. A typical walking stride lasts about one second meaning current methods can only measure the mean metabolic cost following a bout of steady-state walking. Experiments that approximated the cost of the swing phase by recording cyclical leg swinging [14] and by measuring blood flow from injected microspheres in animals that are then sacrificed [15] suggest that the stride-mean metabolic cost does not necessarily represent the contributions of individual phases (‘within-stride metabolic cost’).

Several model-based methods of estimating within-stride metabolic cost have been proposed but remain inconclusive. Umberger developed a set of equations to estimate metabolic cost from muscle parameters and used this to produce the first estimation of within-stride metabolic cost from a forward simulation of walking [16]. Other groups used EMG-driven simulations [17] or equations based on joint kinetics instead of muscle parameters [18]. However, when comparing those methods to each other, their estimations of within-stride metabolic cost are relatively inconsistent (Pearson correlation: *r =* 0.29, *n* = 6 estimations, Fig. 1B) [19]. Currently, there is no way to validate these model-based estimations for within-stride metabolic cost since measurements from indirect calorimetry only obtain a stride mean. This motivates the development of an alternative method to estimate within-stride metabolic cost that is supported by indirect validation approaches.

We hypothesized that applying a set of perturbations creates a set of instances of the behavior where the differences in the time series between each perturbed instance can be attributed to the different magnitudes and timings of the applied perturbation. By applying perturbations repeatedly to a specific part of the gait cycle for several minutes, we can induce changes in the stride-mean metabolic cost as well as in the biomechanical time series (e.g., kinematics, kinetics, and muscle activations) [19–21]. We postulated the variation across the set of perturbed walking strides would be representative of the fluctuations in metabolic cost within the stride cycle so long as the set contained a large number of different perturbations. If true, this would enable a method to extract key features of within-stride metabolic cost. Our approach is inspired by prior studies that utilized ankle perturbations to assess time series of joint impedance during the stance phase [22, 23] as well as studies that used elastic bands and added mass to estimate the cost of stance and swing phases [24, 25]. To the best of our knowledge, using of a perturbation-based approach for estimating within-stride metabolic cost time series is novel.

Using this concept, extraction of within-stride behaviour from a collection of perturbed instances, we developed an alternative method to estimate within-stride metabolic cost that we refer to as our ‘perturbation-based method’. Our method estimates within-stride metabolic cost using measurements from a set of perturbed walking strides. We then evaluated our method’s ability to consistently reproduce model-based estimates of within-stride metabolic cost.

## Materials and methods

### Overview

We created two datasets; a dataset generated using a neuromechanical simulation and a dataset collected from human experiments [20, 26, 27]. Each of these datasets contain walking kinematics, kinetics, and muscle activation time series during 35 different perturbed walking conditions and one unperturbed, normal walking condition (36 walking conditions per dataset). The ‘perturbation’ applied during each of the 35 perturbed walking conditions was a force profile applied to the COM.

Our perturbation-based method was initially developed and tuned using the dataset from the neuromechanical simulation [26, 27]. Tuning consisted of adjusting which kinematic, kinetic, and muscle activation time series from the neuromechanical simulation were used for estimating within-stride metabolic cost. The timeseries included from different combinations of kinematic, kinetic, and muscle activation data will be referred to as ‘derived time series’. The choices from tuning were made based on the perturbation-based method’s performance at estimating other kinematic, kinetic, and muscle activation time series.

We indirectly validated our perturbation-based method by evaluating its ability at estimating 10-model based metabolic cost timeseries. First we inputted the kinematic, kinetic, and/or muscle activation data into 10 model-based methods in order to generate within-stride metabolic cost timeseries (five models per dataset). We then indirectly validated our perturbation-based method based on its ability at reproducing the five model-based metabolic time series from the neuromechanical dataset and the five model-based metabolic time series from the human experimental dataset. Re-evaluating our method in two distinct datasets avoids dataset bias [28]. Finally, we assessed the perturbation-based method’s estimate of within-stride metabolic cost when using $\:\dot{V}{O}_{2}$ and $\:\dot{V}{CO}_{2}$ data from the human experiment.

### Simulation dataset

We adapted a neuromechanical simulation from Song and Geyer to walk under force perturbations from a waist tether [26, 27]. Specifically, we used a two-dimensional variant that restricts motion to the sagittal plane [26]. We simulated perturbations with forward forces applied at the hip of a model with seven rigid segments in Simscape First Generation Multibody (MathWorks, Natick, MA). In this framework, we simulated 32 sinusoidal force profiles with peak timings covering the entire gait cycle and peak forces ranging from 0 to 24% of body weight, three constant force profiles, and an unperturbed walking condition.

The neuromechanical model’s walking control strategy was optimized for each perturbed walking condition (cf. Supplementary: Neuromechanical simulation dataset for tuning and in silico evaluation). Briefly, the cost function was piecewise with two different functions based on a conditional of a walking pattern that reached 20 s without a fall. Firstly, the optimization would search for control parameters that increased the distance travelled with consistent stepping, the number of steps taken, and the time the simulation successfully walked without falling. Once a set of control parameters achieved a walking pattern that could walk for a simulated 20 s, the control parameters would be refined to match a target walking velocity of 1.25 ms^− 1^, minimize muscle activations, maintain a consistent walking pattern, and penalize unnatural range of motion.

Time series data (simulated kinematics, kinetics, and muscle activations) were extracted for each of the optimized control strategies to constitute the neuromechanical dataset. We then constructed 100 time series to serve as test data for tuning our perturbation-based method. These test time series were random linear combinations of the different biomechanical time series, so they were distinct from the model-based estimates that would be used later for evaluation.

### Experimental dataset

We used biomechanical and indirect calorimetry data from previous human experiments [20] with a robotic waist tether [21] for the in vivo evaluation and application of our perturbation-based method (Supplementary Data 1). Ten healthy participants (age: 28.0 ± 4.7 years, body mass: 83.2 ± 12.2 kg, height: 1.80 ± 0.05 m; mean ± SD) walked under the same perturbations as in the neuromechanical simulation dataset. In this case, the perturbations were generated by a robotic waist tether controlled by a temporal algorithm that enables pulling during a specific portion of the gait cycle with high consistency.

### Perturbation-based method input signals

**Fig. 2:**
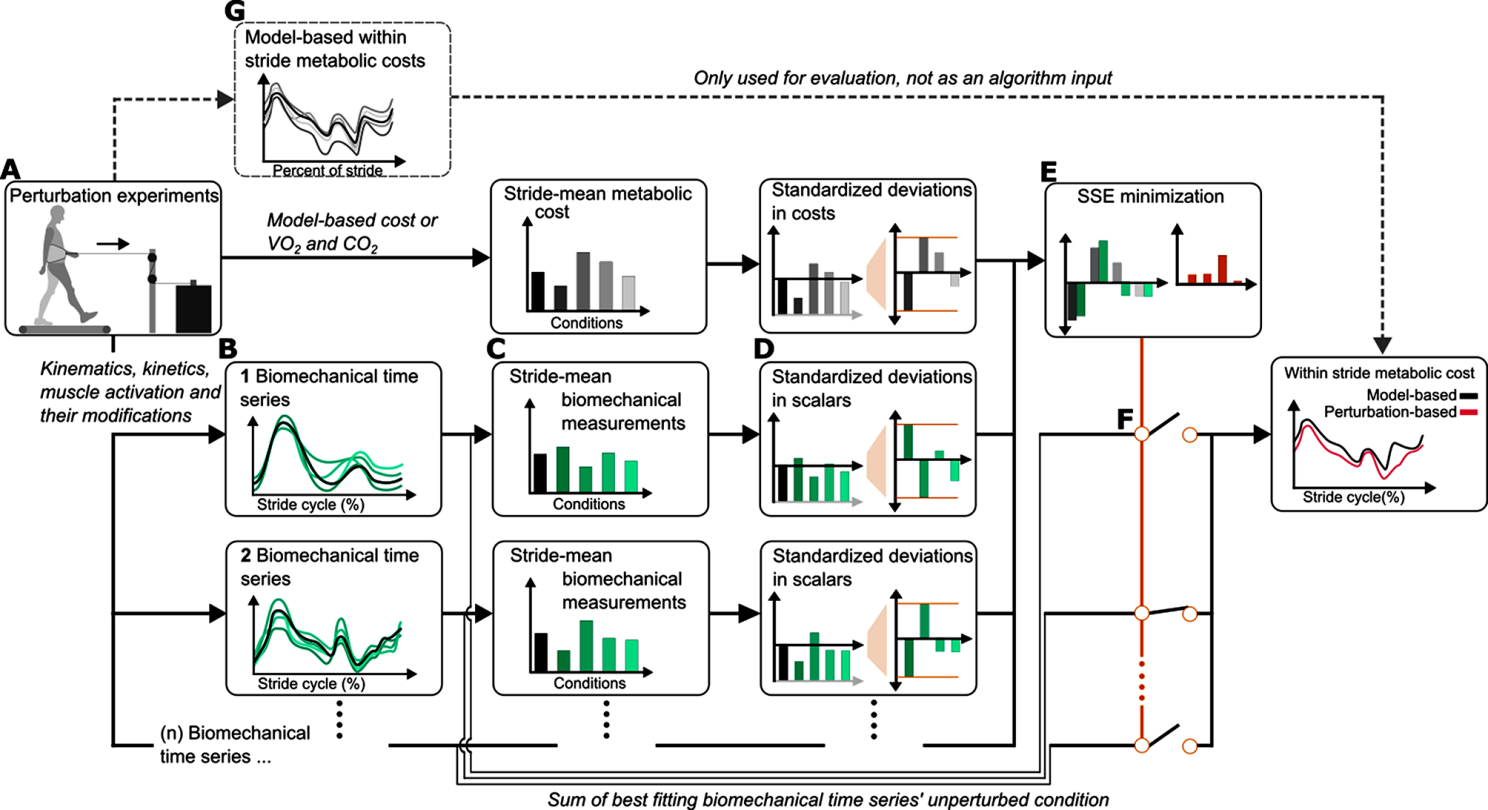
Flow of data for estimating and evaluating within-stride metabolic cost. (**A**). A perturbed dataset was gathered using force perturbations at the COM. Biomechanical time series (e.g., kinematics, kinetics, muscle activations) as well as stride-mean metabolic cost were measured for each walking condition. (**B**). These measurements are stride normalized and **(C).** then converted to a stride-mean for each walking condition. (**D**). The stride means for each biomechanical measurement are custom standardized by subtracting the unperturbed stride mean from each perturbed stride mean and then dividing by the range of deviations from unperturbed walking. (**E**). The custom standardized biomechanical time series are then compared to the custom standardized within-stride metabolic cost using the sum of square error. This process will be iterative, where an additional custom standardized biomechanical time series may be added if it reduces the sum of square error. (**F**). The biomechanical time series or combination of biomechanical time series that corresponded to the lowest sum of square error are selected. The unperturbed condition from the selected biomechanical time series is used as the estimate for within-stride metabolic cost. (**G**). The original model-based within-stride metabolic cost is only used for validation of our perturbation-based method. Our perturbation-based method leverages information from stride-mean values that are experimentally available to indirect calorimetry

Our perturbation-based estimation method uses the stride-mean metabolic cost as well as within-stride biomechanical time series to estimate within-stride metabolic cost (Fig. 2C and F. Methods: Perturbation-based method). The biomechanical time series as well as additional mathematically derived combinations of those time series are considered potential estimates of within-stride metabolic cost (cf. Methods: Additional input signals and algorithm tuning). Our perturbation-based method first calculates the mean cycle from 0 to 100% of the stride for each biomechanical time series for each perturbation condition. Then each stride-normalized biomechanical time series is reduced to one scalar for each perturbation condition using a custom standardization method based on the deviation from unperturbed walking (Fig. 2, cf. Methods: Custom standardization method). A collection of these standardized scalar values of biomechanical data across all perturbations form a perturbed biomechanical set. Finally, we select the biomechanical set that matches the perturbed set of the stride-mean metabolic cost (cf. Methods: Time series estimation procedure). The original biomechanical time series that most closely matched the standardized set for the stride-mean metabolic cost is used as the estimate of within-stride metabolic cost.

We chose to estimate the metabolic cost of one side of the body rather than the whole body’s metabolic cost. The within-stride metabolic cost of one side of the body provides more descriptive and potentially useful information for interventions, such as assistive devices, than whole-body cost, which cannot be attributed to a specific leg. Using model-based methods, we generated a set of five estimates of the within-stride metabolic cost to indirectly validate our perturbation-based method’s performance which were distinct from the five evaluations that were used in the neuromechanical dataset (cf. Supplementary: Model-based metabolic costs used; [18, 29–35]).

All kinematic, kinetic, and muscle activation time series as well as the derived signals (cf. Methods: Additional derived input time series and algorithm tuning) are stride-normalized and organized in matrices with one row for each percent of the stride cycle and one column for each of the 36 perturbation conditions.

1 $$\:{X}_{bts}=\left[100\:\times\:\:36\right]$$

Each perturbation’s force profile was repeated over multiple stride cycles for a sufficient duration to obtain steady-state metabolic cost (40 s to obtain ten sufficiently stable strides in the neuromechanical simulations and 2 min to estimate the steady-state metabolic cost in the human experiments) [11].

The stride mean metabolic cost for every condition is also used as an input in our perturbation-based method.

2 $$\:\stackrel{-}{Y}=\left[1\:\times\:\:36\right]$$

This stride mean can be estimated from model-based metabolic costs as well as from respiratory $\:\dot{V}{O}_{2}$ and $\:\dot{V}C{O}_{2}$ measurements; hence this input is available when estimating the within-stride metabolic cost in human experiments.

### Custom standardization method

Each time series is standardized using a custom method (Supplementary Data 1). First, we take the stride mean of each biomechanical time series for every perturbation condition.

3 $$\:{\stackrel{-}{X}}_{bts}=\left[1\:\times\:\:36\right]$$

Next, we calculate the deviation of each perturbed walking condition from the unperturbed walking condition.

4 $$\:{\Delta\:}{\stackrel{-}{X}}_{bts}={\stackrel{-}{X}}_{bts}-{\stackrel{-}{X}}_{bts,0}\:=\left[1\:\times\:\:36\right]$$

where $\:{\Delta\:}{\stackrel{-}{X}}_{bts}$ is the set of deviations from the unperturbed condition and $\:{\stackrel{-}{X}}_{bts,0}$ is the stride mean of the unperturbed condition.

Each set of deviations is then normalized by its range of deviations from unperturbed walking.

5 $${\mathop X\limits^ - _{stand}}\> = round\left( {{{{{\mathop {\Delta X}\limits^ - }_{bts}}{n_{bins}}} \over {max\left( {{{\mathop {\Delta X}\limits^ - }_{bts}}} \right) - {\rm{min}}\left( {\Delta \>{{\mathop X\limits^ - }_{bts}}} \right)}}} \right) = \left[ {1\> \times \>\>36} \right]$$

where $\:{\stackrel{-}{X}}_{stand}\:$is the standardized set of deviations from unperturbed walking for each biomechanical time series and n_bins_ is the number of bins. The standardized set is enumerated to reduce the effects of floating-point differences between biomechanical measurements. The number of bins was set to 80 based on tuning (cf. Methods: Tuning of available data for metabolic cost estimation, Supplementary Data 2). This process is similar to Slade et al., (2022) [36].

In summary, this procedure converted the stride means of biomechanical time series to a range of standardized values ranging from 1 to 80. We also applied the same standardization procedure (Eqs. 4, 5) to the stride means of derived biomechanical time series as well as to the stride mean metabolic cost ($\:\stackrel{-}{Y}$).

### Time series estimation procedure

We ran a minimization procedure that evaluates which standardized biomechanical time series best matches the standardized metabolic cost. First, we evaluate how well the standardized set of each biomechanical time series and each derived time series matches the standardized set of metabolic cost using a sum of square comparison

6 $$\:{SS}_{initial}={\sum\:}_{c\:=\:1}^{cond\:36}{\left({\stackrel{-}{X}}_{stand,\:c}-\:{\stackrel{-}{Y}}_{stand,c}\right)}^{2}\:$$

where $\:SS$ is the sum of squares and $\:c$ represents each perturbation condition.

Then, we conduct a stepwise optimization procedure whereby we evaluate if adding another standardized biomechanical time series or derived signals to the previous standardized set improves the $\:SS$

7 $$\eqalign{& \>S{S_{new}} = \>\sum {\>_{c\, = \>1}^{cond35}} \cr& \quad {\left( {\left( {{{\mathop X\limits^ - }_{stand,c,j}} + \>{{\mathop X\limits^ - }_{stand,c,\>\>prev\>opt\>SS}}} \right) - \>{{\mathop Y\limits^ - }_{stand,c}}} \right)^2} \cr}$$

where $\:{\stackrel{-}{X}}_{stand,c,\:prev\:opt\:SS}$ is the standardized set that produced the best SS in the previous iteration and *j* represents a new biomechanical measurement or derived signal that is evaluated.

**Fig. 3:**
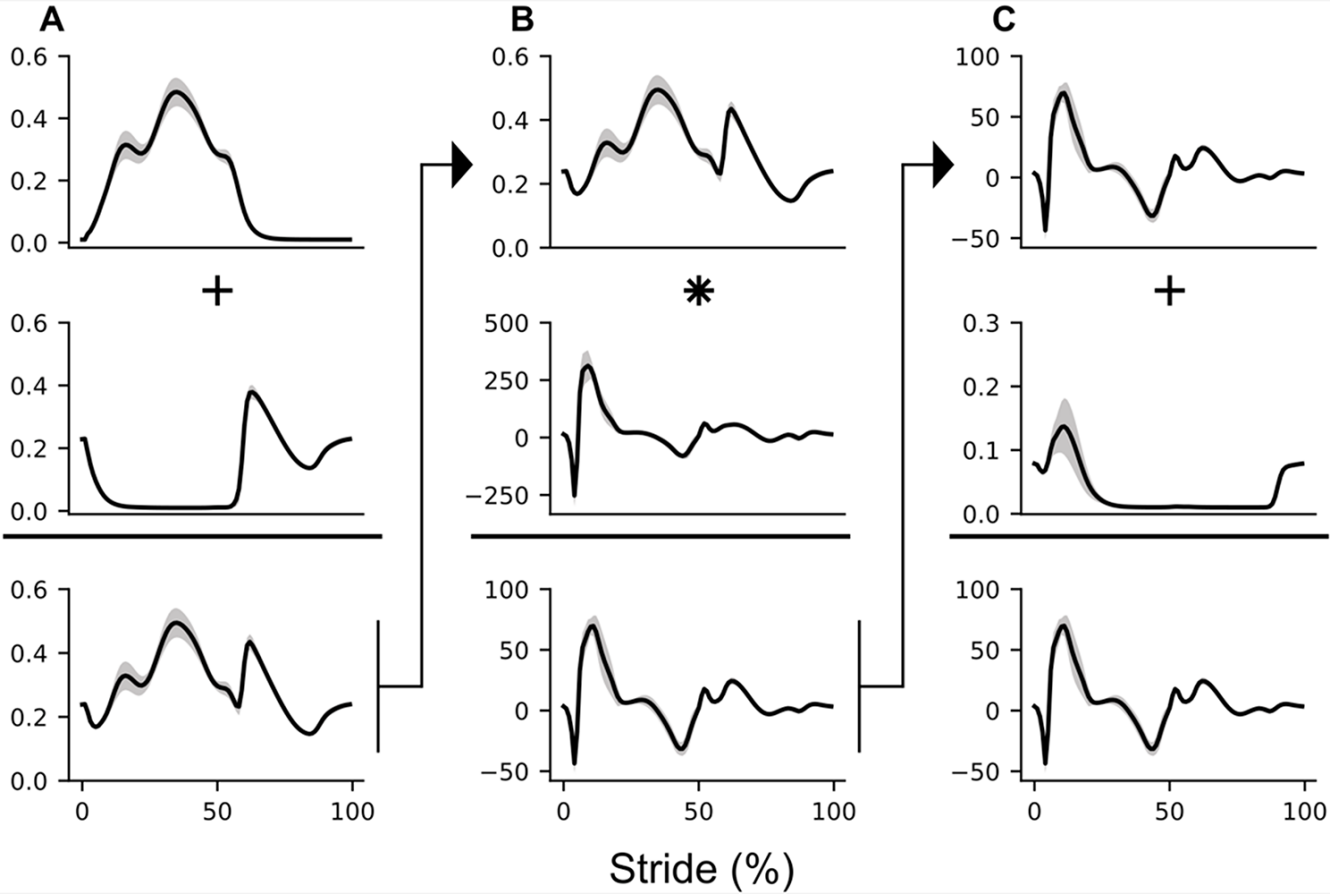
Illustration of how biomechanical and derived time-series are combined to produce a within-stride metabolic cost time series. Each column (**A**, **B**, **C**) in the figure represents a mathematical operation used to create a new time series. The final plot on the bottom right is the estimated within-stride metabolic cost. The specific combination shown here was used to estimate the Bhargava et al., 2004 metabolic cost in Table 1

Finally, the time series of the biomechanical measurement, derived signal, or combination of signals with the lowest $\:SS$ is then used to estimate within-stride metabolic cost (Fig. 3). If the lowest SS results from one single biomechanical measurement or derived signal, the corresponding unperturbed time series is used to estimate within-stride metabolic cost.

8 $$\:{Y}_{estimated}={X}_{SS\:opt}=\:\left[100\:\times\:\:1\right]$$

where $\:{Y}_{estimated}$ is the estimated within-stride metabolic cost, $\:{X}_{SS\:opt}$is the time series of the biomechanical measurement or derived signal that resulted in the lowest $\:SS$. In the event the lowest $\:SS$ is from a combination of biomechanical measurements and derived signals, we normalize each signal by its range and sum to serve as the estimate of within-stride metabolic cost

9 $$\eqalign{{Y_{estimated}} & = \>\sum \> _{i\, = \,1}^{number\>of\>bts} \cr & {{{X_{bts\>\>SS\>opt,\>i}}} \over {{\rm{max}}\left( {{X_{bts\>\>SS\>opt,i}}} \right) - {\rm{min}}\left( {{X_{bts\>\>SS\>opt,i}}} \right)}} \cr}$$

where $\:i$ is the index of the biomechanical signals used to achieve the lowest sum of squares.

The approach of leveraging perturbations constitutes a paradigm shift compared to previous iterative improvements of model-based methods. Our procedure of using data from the perturbed conditions to estimate the unperturbed condition intrinsically involves estimating (just) outside of test data, and it is known that overfitting can be an issue in such a procedure. Some features of the perturbation-based method likely helped avoid this overfitting. We limited the number of inputs by using a standardization that converted each time series to a scalar (Eqs. 3–5). We also generated a very large number of derived signals.

### Additional derived input signals and algorithm tuning

We tuned two features of our perturbation-based method: the selection of which mathematical derived time series would be available for creating the estimation of within-stride metabolic cost and the number of bins in the custom standardization procedure (cf. Methods: Additional derived input signals and algorithm tuning). During the tuning, we evaluated which settings improved the lower-bound, 95% confidence interval of Pearson’s correlations between the estimated and the test time series. After tuning, the mean Pearson’s correlation between our perturbation-based method’s estimate and time series within the test set was 0.41 (95% CI = 0.33–0.50). We evaluated the impact of the following options:

- Options 1–2: The separation of positive and negative regions of the original biomechanical time series.
- Options 3–5: The square, cube, or inverse of the original biomechanical time series.
- Options 6–8: The subtraction, addition, or multiplication of all pairs of the biomechanical time series.
- Option 9: An additional set of additions and multiplication of pairs of the mathematically derived time series (generated from options 1–8).

We restricted option 9 to stop after generating 4000 combinations because considering all the combination permutations was not feasible. We also tuned the number of bins for standardizing biomechanical time series (Eq. 5). This tuning is similar to the sensor selection and bin optimization in Slade et al. [36].

The tuning criterion was correlation performance against 100 test time series. The test time series used were distinct from the model-based metabolic costs to avoid biasing the evaluation of our method [28]. As test time series for tuning, we generated 100 time series based on random combinations of the biomechanical time series from the neuromechanical simulation dataset.

10 $$\eqalign{ \>{Y_{tuning,k}} = & \>{c_1}\left| {{X_{bts,1}}} \right|\> + \>{c_2}\left| {{X_{bts,2}}} \right| \ldots \>\> \cr & {c_n}\left| {{X_{bts,n}}} \right| = {\rm{[}}100 \times \>36{\rm{]}} \cr}$$

where $\:{Y}_{tuning,k}$ represents one of the 100 test time series,$\:\:{c}_{1}$ to *n* are random coefficients between 0 and 1, $\:{X}_{bts,1}$ to $\:{X}_{bts,n}$ are the positive or negative portions of a randomly chosen number of biomechanical measurement time series.

The perturbation-based method’s correlation with the 100 test time series was evaluated for each of 512 (2^9^) combinations of mathematically derived time series for bin numbers ranging from 10 to 100 (Supplementary Data 2).

### Statistical analysis

As a measure of the uncertainty in the literature, we generated a cross-table with pairwise Pearson correlations between six previously reported plots of within-stride metabolic cost in the literature [19], and we calculated the mean and 95% confidence interval of the correlations (Fig. 1b). Due to the limits of a Pearson correlation at − 1 and 1, we converted each *r*-value to a Z-score using Fisher’s Z-transformation. Average Z-scores and z-score confidence intervals across the correlations in literature, between perturbation-based and neuromechanical model-based, and between perturbation-based human experimental model-based were converted back to Pearson *r*-values for easier interpretation [37]. All analyses were conducted in MATLAB 2021b.

## Results

**Fig. 4:**
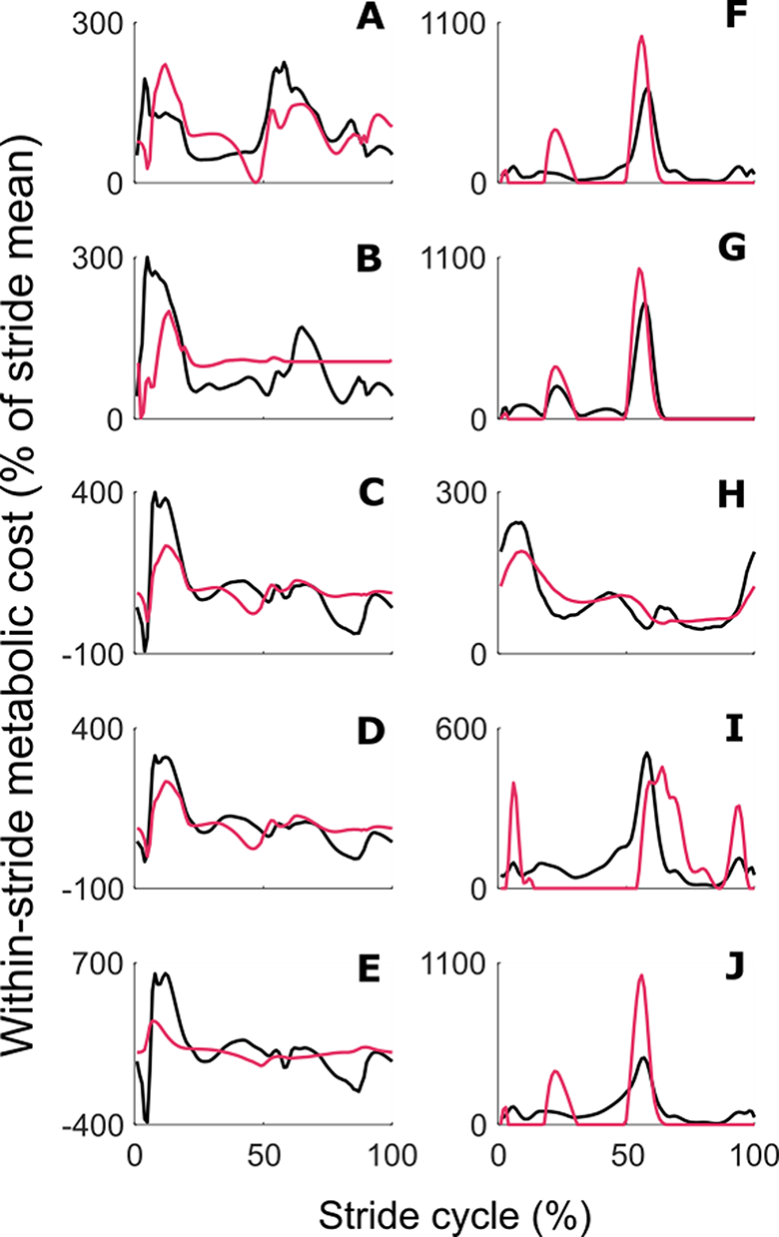
Evaluation of perturbation-based method. Evaluation of perturbation-based method’s ability to reproduce within-stride metabolic cost of different model-based methods in our two datasets. Estimations from each model-based method are represented by black lines. Our perturbation-based method’s estimations are represented with red lines. The left column shows evaluations in the neuromechanical simulation dataset, and the right column shows evaluations in the human experiment dataset. (**A**). Umberger et al., 2003 [35], (**B**). Houdijk et al. 2006 [33], (**C**). Bhargava et al., 2004 [32], (**D**). Lichtwark et al., 2005 [34] (**E**). Margaria 1968, applied onto muscle work rate [30, 52], (**F**). Beck et al., 2019 [31], (**G**). Kim and Roberts, 2015 [18] (**H**). Margaria 1968, applied onto COM work rate [30, 53], (**I**). Margaria 1968, applied onto joint work rate [30, 54] (**J**). Minetti and Alexander, 1997 [29]

Once tuning was completed, and our perturbation-based method was finalized, we evaluated its performance at reproducing a variety of model-based estimates of within-stride metabolic cost. We calculated five within-stride metabolic costs using model-based methods (cf. Supplementary: Model-based metabolic costs used in neuromuscular simulation dataset; [30, 32–35]). The mean Pearson’s correlation between the five different model-based within-stride metabolic costs and our estimations of those using the perturbation-based method was 0.55 (95% CI = 0.22–0.77). This evaluation performance constitutes an improvement of at least 50% compared to the mutual consistency between model-based estimations in the literature for four out of five estimations (Fig. 4A-E; Table 1).

**Table 1:** Evaluation of perturbation-based method in neuromechanical dataset

Stride mean metabolic cost input	Selected mathematically derived combination of biomechanical time series	Estimated versus actual time series correlation ^┼^
Bhargava et al., 2004	(Soleus + tibialis anterior) * hip power + vastus medialis	0.76
Houdijk et al., 2006	(COM power + vastus medialis) * rectus femoris + vastus medialis	0.22
Lichtwark et al., 2005	(Soleus + tibialis anterior) * hip power + vastus medialis	0.77
Margaria,1968, muscle-based	Knee angle – hip moment	0.49
Umberger, 2003	(Stride time + vastus medialis) * hip power + vastus medialis	0.42
**Mean Pearson correlation 0.55 (95% CI = 0.22–0.77)** ^*^		

┼ The final column lists correlations between model-based within-stride metabolic costs and estimations of these costs using the perturbation-based method (Fig. 4 AE). The stride mean metabolic costs used as inputs for the perturbation-based estimation are named in the first column. The Pearson correlations serve as a measure of the estimation performance

* Mean Pearson correlation and confidence interval are calculated following Fisher Z transformation

We also indirectly validated our perturbation-based method in data from human experiments. In vivo, human walking experiments were conducted with a perturbation from a robotic waist tether applied to the COM (cf. Supplementary: Human experimental dataset for in-vivo evaluation and application) [20]. In each condition, the tether applied pulling forces with a specific profile repeatedly to stride cycles for a sufficient duration to induce a different steady-state gait. We applied the same perturbation-based method to our human experimental dataset without any additional tuning or changes. Our estimation reproduced the above-mentioned five independent model-based estimations of metabolic cost with a mean Pearson’s correlation of 0.80 between the model-based metabolic costs and their estimations using the perturbation-based method (95% CI = 0.57–0.91, Table 2). This result is also greater than the correlation between model-based estimations currently in literature with an improvement of at least 75% (Fig. 4F-J) [19, 38].

**Table 2:** Evaluation of perturbation-based method in human experiment dataset

Stride mean metabolic cost input	Selected mathematically derived combination of biomechanical time series	Estimated versus actual time series correlation ^┼^
Beck et al., 2019	Hip angle – vastus medilias + gluteus maximus + vertical GRF	0.86
Kim and Roberts, 2015	(Positive portion of hip power)	0.41
Margaria, 1968 COM-based	(COM power positive portion) * soleus + vertical GRF	0.91
Margaria, 1968 joint-based	(COM power positive portion) * vastus medialis + vertical GRF	0.78
Minetti and Alexander, 1997	(COM power positive portion) * tibialis anterior + vertical GRF	0.83
$\:\dot{V}{O}_{2}$ and $\:\dot{V}C{O}_{2}$	Hip angle – tibialis anterior + gastrocnemius + vertical GRF	N/A ^#^
**Mean Pearson correlation 0.80 (95% CI = 0.57–0.91)** ^*^		

┼ The final column lists correlations between model-based within-stride metabolic costs and estimations of these costs using the perturbation-based method (Fig. 4 FJ). The stride mean metabolic costs used as inputs for the perturbation-based estimation are named in the first column. The Pearson correlations serve as a measure of the estimation performance

* Mean Pearson correlation and confidence interval are calculated following Fisher Z transformation

# The final row shows the combination that was selected to plot the within-stride metabolic cost time series based on respiratory V̇O_2_ and V̇CO_2_ data. In this application, there was no reference to compare our estimation-performance against; hence no correlation is reported

**Fig. 5:**
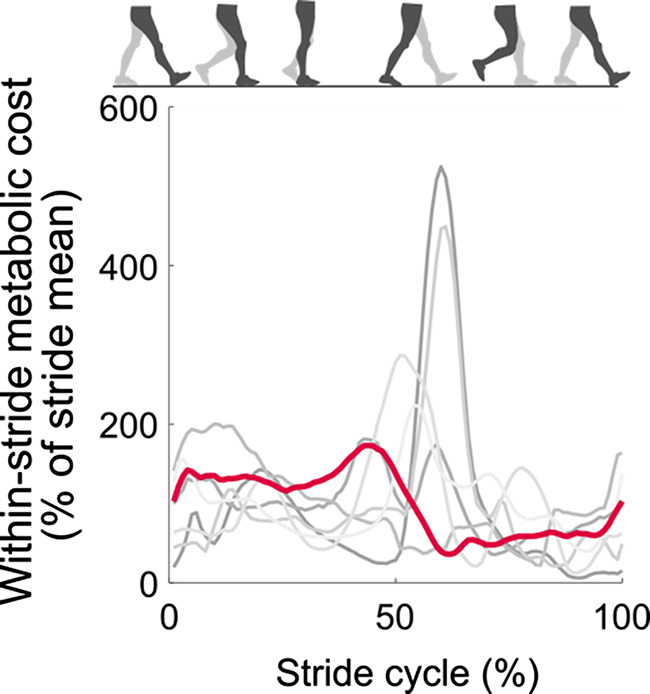
Application of the perturbation-based method to estimate within-stride metabolic cost. The red line shows the perturbation-based estimate of within-stride metabolic cost using stride means of $\:\dot{V}{O}_{2}$ and $\:\dot{V}{CO}_{2}$ from the human experiment dataset as inputs. The grey lines show previous estimations from model-based methods which have previously been published in the literature [19].

After successfully completing the indirect validations, we applied our perturbation-based method to estimate within-stride metabolic cost based on $\dot V{O_2}$ and $\dot VC{O_2}$ data from the human experiment (Fig. 5). When we divide the stride into the first double stance (1–15% of the stride), single stance (16–50%), push-off (51–65%), and swing (66–100%), their metabolic cost respectively accounted for 20, 49, 10 and 21% of the total. The estimated cost of push-off is considerably lower than that of single stance. This is markedly different from the evolution of positive mechanical work performed by the leg onto the COM, which is about three times as much during push-off compared to single stance. As such, our perturbation-based estimation confirms that metabolic cost can be related to sources other than mechanical work [39, 40].

Our estimation that push-off accounts for about one-tenth of the total metabolic cost is similar to the first estimation using a forward-dynamics musculoskeletal model-based approach (8% [16]) but is low compared to estimations from model-based methods that use only joint-based equations (39% [18] and 49% [19]). Our estimation of the cost of the swing phase (21%) is close to the mean from previous model-based studies (24%, 95% CI = 19–28% [16, 17, 19, 38, 41, 42]). This also supports previous estimations from experimental studies with perturbations to the swing or stance phase that suggest that the swing phase substantially contributes to the metabolic cost of walking (swing phase contribution to metabolic cost reported as 10, 12.5 and 17% [14, 24, 25]).

While our approach of using perturbations is innovative and yields results consistent with existing literature, we acknowledge some limitations in our methods, results, and the application. One methodological limitation is that our method solely relied on lower limb signals for estimating metabolic costs. Our evaluation replicated model-based costs using lower-limb data and a simplified neuromuscular model. Notably, we did not directly account for metabolic contributions from trunk and arm muscles [43]. Another methodological constraint is the tuning of the derived time series and the number of perturbations required to create the datasets. Adapting this method for other datasets might require expanding the types of derived time series. In terms of the results, we recognized that our perturbation-based method for estimating within-stride metabolic cost is empirical. While this offers the advantage of being less biased than model-based methods, this is not favorable for understanding causal relationships, such as the impact of altering a specific gait impairment [27, 44, 45]. Application-wise, a drawback of our method is its reliance on datasets of walking under various perturbations which can be time-consuming and physically demanding for participants.

To advance perturbation-based within-stride metabolic cost estimation’s practicality, future research needs to tackle challenges concerning tuning, time efficiency, and validation. Developing algorithms with greater generality, such as neural networks, could mitigate reliance on specific tuned options. Investigating perturbation types yielding the most valuable data will streamline data collection efforts. Finally, exploring innovative indirect validation methods could bolster confidence in the methodology.

## Conclusion

The present work describes a perturbation-based method that can reproduce a wide variety of model-based, within-stride metabolic costs in two different datasets using a collection of perturbed conditions. The result suggests that the metabolic cost of push-off is lower than the preceding single stance phase and that the swing phase has a non-negligible metabolic cost. These findings may have important applications for designing rehabilitation strategies and assistive devices. For example, the finding of a large cost of single stance may help explain how an unpowered ankle exoskeleton that primarily provides torque during single stance could reduce metabolic cost despite increasing plantar flexor activation during push-off [46]. The trajectory of community research has incrementally reduced the time to estimate steady-state metabolic cost from several minutes using Douglas bag, mixing chamber, to 1–2 min with breath-by-breath systems [47] and fitted approximation methods [11, 48, 49], and finally, to a matter of seconds via a combination of sensors and fitting methods [50, 51]. The present work grants greater understanding of metabolic cost beyond what was previously possible by presenting within movement cycle interpretability instead of more rapid interpretation of steady-state metabolic cost.

### Electronic supplementary material

Below is the link to the electronic supplementary material.

Supplementary Material 1

Supplementary Material 2

Supplementary Material 3

## Data Availability

All data are available in the main text or the supplementary materials.

## Abbreviations

EMG: Electromyography
COM: Center of Mass
