# Supplementary material for: Perturbation-based estimation of within-stride cycle metabolic cost
Source: J Neuroeng Rehabil. 2024 Aug 1;21:131. doi: 10.1186/s12984-024-01424-8 (PMC11293076; doi:10.1186/s12984-024-01424-8)
Supplement: Supplementary file 3 — Supplementary Material 3 [file 12984_2024_1424_MOESM3_ESM.docx]

**Supplementary methods**

Neuromechanical simulation dataset for tuning and in silico evaluation

We adapted a neuromechanical simulation from Song and Geyer to walk under force perturbations from a waist tether [1,2]. Specifically, we used a two-dimensional variant that restricts motion to the sagittal plane [1]. We simulated perturbations with forward forces applied at the hip of a model with seven rigid segments in Simscape First Generation Multibody (MathWorks, Natick, MA). In this framework, we simulated 32 sinusoidal force profiles with peak timings covering the entire gait cycle and peak forces ranging from 0 to 24% percent of body weight, three constant force profiles, and an unperturbed walking condition. These conditions were based on previous human experiments, which ensures that the perturbations for estimating the within-stride metabolic cost are feasible [3]. The model is actuated by nine Hill-type muscles per leg that are controlled by 71 adjustable parameters representing muscle-reflex pathways divided among 10 spinal modules. The segment and muscle parameters are based on Song and Geyer (2015) [1].

We minimized a cost function based on physiologically inspired objectives and experimental conditions to select an optimal walking strategy under each perturbation condition. Minimizing the cost function was designed to produce realistic walking strategies that maintained a speed close to the target speed (1.25 ms^-1^) with the least amount of effort for 20 s.

$J=\left\{ \begin{aligned} 2000-d_{hip}+x_{steady}-t-n_{steps} if fall \\ 100\left\| v_{tgt}-v_{mean} \right\|+x_{steady}+pain+C_{E} if walk \end{aligned} \right\}$ *(11)*

where $J$ is the cost function, $d_{hip}$ is the distance traveled by the pelvis, $x_{steady}$ is the distance traveled in an unstable stepping pattern, $t$ is the time before a fall, $n_{steps}$ is the number of steps taken, $\left\| v_{tgt}-v_{mean} \right\|$ is the difference between the mean walking velocity and the target velocity, $pain$ is a penalty function designed to avoid unnatural joint overextension, and $C_{E}$ is muscle effort. Muscle effort is

$C_{E}=\sum_{m=1}^{muscle 9} \int\frac{{A_{m}}^{2}}{d_{hip}}dt$ *(12)*

where $A_{m}$ is the simulated muscle activation of the 9 muscles in the model [4,5]. In summary, the first goal of the cost function (eq. 11) was avoiding a fall. Following a walk that successfully reached 20 s, the strategy was refined to maintain a consistent walking pattern, maintain a target speed, minimize effort, and avoid joint overextension.

Covariance Matrix Adaptation Evolution Strategy (CMA-ES) was used as the optimization algorithm to search for muscle control parameters that minimized the cost function [6]. CMA-ES relies on a multivariate normal distribution that uses the previous best solutions to update the covariance matrix, mean, and standard deviation of the control parameter space. We used optimized muscle control parameters for normal walking, as reported by Song and Geyer (2015), as the initial guess, as well as an initial population of 16 and a standard deviation of 0.05 [1].

We ran all calculations using a super-computing cluster (Holland Computing Center, University of Nebraska Lincoln). Optimization run times were limited to 90 hours, and each perturbation condition was successfully optimized at least four times. A successful optimization was considered a cost function value of less than 20. In the event the CMA-ES algorithm did not achieve a cost function value of less than 20 in under 90 hours, the optimization process was restarted up to 10 additional times until at least four optimized sets of control parameters were obtained.

Human experimental dataset for in-vivo evaluation and application

We used biomechanical and indirect calorimetry data from previous human experiments [3] with a robotic waist tether [7] for the in vivo evaluation and application of our perturbation-based method (Supplementary Data 1). Ten healthy participants (age: 28.0 ± 4.7 years, body mass: 83.2 ± 12.2 kg, height: 1.80 ± 0.05 m; mean ± SD) walked under the same perturbations as in the neuromechanical simulation dataset. In this case, the perturbations were generated by a robotic waist tether controlled by a temporal algorithm that enables pulling during a specific portion of the gait cycle with high consistency.

The type of perturbation with timed forces at the centre of mass was likely more beneficial for extracting within-stride metabolic cost than constant perturbations such as changes in treadmill speed or inclination. Pilot analyses with earlier algorithms using a dataset that involved modifications in treadmill and footwear inclination [8] suggest that the type of perturbation matters. By applying perturbations at the centre of mass, we could obtain relatively large changes in biomechanics and metabolic cost (48% in the human experiments) compared to the lower changes that are typically obtained with other wearable robots. The large change in metabolic cost itself likely aided the perturbation-based method’s performance by widening the differences between stride-mean metabolic cost used during within-stride estimation (eqs. 6-9). Furthermore, by using only assistive pulls as the type of perturbations, the opportunity for human data collection may expand to patient populations that are unable to walk for longer durations. However, there is still most likely a minimum required number of perturbations needed to generate a within-stride estimation. Still, a minimum number of perturbations would mean a minimum amount of walking time required for within-stride estimation, which may exclude especially walking-impaired patient populations. To address this concern, future experiments could identify the most effective perturbations, which may reduce the total number of perturbations required to estimate within-stride metabolic cost.

We measured joint kinematics (14-camera motion capture system; VICON Vero, Oxford Metrics, Yarnton, UK; 100 Hz), ground reaction forces (GRF; Bertec, Columbus, OH, USA; 1000 Hz) and muscle activation of the right leg using a wireless electromyography (EMG) system (Trigno TM, Delsys, USA; 2000 Hz) as well as respiratory O_2_ consumption and CO_2_ production (K5, Cosmed, Rome, Italy). We filtered the marker data and GRF data with a 10Hz low-pass Butterworth filter and calculated angles, moments, and powers in Visual3D (C-Motion, Germantown, MD, USA). We processed EMG data with a high-pass filter with a 20Hz cut-off frequency, followed by rectification, and a low-pass filter with a 6Hz cut-off frequency. We detected EMG artefacts by inspecting how well signals stayed within a band of 1.5 times the interquartile range and qualitatively removed extreme outliers. All estimations using our perturbation-based method were conducted using the mean stride of all the participants in each perturbation condition. Additional details on the respiratory data processing and protocol can be found in Antonellis et al., 2022 [3].

Evaluation of data-driven method

We evaluated our perturbation-based method's ability to reproduce different model-based estimates of within-stride metabolic cost. Using model-based methods from literature, we generated different within-stride metabolic cost time series under each perturbed walking condition and normalized to a percent of stride resulting in the following matrix dimensions [9–18].

$Y_{actual}=[100 \times36]$ *(13)*

where $Y_{actual}$ represents one model-based method of within-stride metabolic cost which the perturbation-based method tries to reproduce. The columns are the 36 perturbed walking conditions.

Our perturbation-based method uses the stride mean metabolic cost of each condition as one of the inputs to generate an estimate of within-stride metabolic cost.

$\bar{Y}_{actual}=[1 \times36]$ *(14)*

where $\bar{Y}_{actual}$ represents the stride mean of a model-based metabolic cost.

For each model-based metabolic cost, we calculated the Pearson correlation coefficient between the actual and estimated within-stride metabolic cost produced by each model-based method and our perturbation-based method, respectively. Our perturbation-based method was blinded to the time series of model-based metabolic costs. Instead, the time series of model-based within-stride metabolic costs were only used at the end to evaluate the performance of our perturbation-based method. We report the mean and 95% confidence interval from the validation tests of our method to serve as an estimation of the level of confidence that is to be expected when estimating within-stride metabolic cost from respiratory methods

We evaluated our method using five different model-based metabolic costs for the musculoskeletal simulation dataset and five different model-based metabolic costs for the human experiment dataset. This number of evaluations was not based on a statistical power analysis, but we rather tried to use the maximum available number of model-based metabolic costs by including both ones that are commonly cited [10,19] or have been explicitly used for estimating within-stride metabolic cost [9,11] in addition to ones that have been used only rarely [20], or ones that use simple measurements and are only expected to coarsely estimate metabolic cost [15]. In a previous publication, we gathered all available literature sources on model-based estimation of within-stride metabolic cost [7]. Some of these estimations of within-stride metabolic cost were provided with the publication [11,21], and others were derived from published supplementary data [22] or calculated using our own motion-capture data [23]. The methods for obtaining the data from these literature sources are described in more detail in our group’s previous work, Gonabadi et al. [23]. We did not include the data from Markowitz and Herr (2016) [24] since they only reported the mean of the two double stance phases, which prevents reconstructing how metabolic cost varies between the first double stance phase and push-off. In addition to the estimations previously reported in Gonabadi et al. [23], we also obtained one additional within-stride metabolic cost from a more recent publication [25]. From that publication, we added the mean within-stride metabolic costs from their normal walking condition.

As a clarification, the evaluation of our method does not rely on the model-based methods to be accurate estimations of metabolic cost. Since our method is not specifically tuned for estimating within-stride metabolic cost but instead tuned to be generalizable, the purpose of the evaluations is to test if our method can consistently reproduce a wide variety of model-based metabolic costs. To maximize this variety in model-based metabolic costs and minimize dataset bias, we used five different model-based metabolic costs in each of the two datasets.

Model-based metabolic costs used in neuromuscular simulation dataset

*Bhargava et al., 2004.* This model used the activation, fibre force, fibre length, and fibre contraction velocity of the simulated muscles of the right leg as inputs [13]:

$Y_{Bhargava}= \sum_{m=1}^{muscle 9} \dot{h}A_{m}+ \dot{h}M_{m}+ \dot{h}S_{m}+ \dot{w}_{m}$ *(15)*

where $\dot{h}A_{m}$, $\dot{h}M_{m}$, and $\dot{h}S_{m}$ are the metabolic costs due to energy losses from heat associated with activation, maintenance, and shortening of the simulated muscles, respectively. $\dot{w}_{m}$ is the metabolic cost due to positive and negative work rates of the simulated muscle fibers calculated from fiber contraction velocity and fiber force. We used both the metabolic cost model as well as the orderly recruitment model for estimating excitation, similar to the implementation in Koelewijn et al. (2019) [10]. We generated the within-stride metabolic cost of the right leg by calculating the sum of all the metabolic cost components of the right leg muscles.

*Houdijk et al., 2006.* This model used the activation, fibre force, fibre length, and fibre contraction velocity of the simulated muscles of the right leg as inputs [12]:

$Y_{Houdijk}= \sum_{m=1}^{muscle 9} \dot{h}A_{m}+ \dot{h}M_{m}+ \dot{h}{SL}_{m}+ \dot{w}_{m}$ *(16)*

where $\dot{h}M_{m}$ is the maintenance heat rate and $\dot{h}{SL}_{m}$is the metabolic cost due to heat losses due to both the lengthening and shortening of muscle fibers.

*Lichtwark et al, 2005.* This model used the fibre force, fibre length, and fibre contraction velocity of the simulated muscles of the right leg as inputs [14]:

$Y_{Lichtwark}= \sum_{m=1}^{muscle 9} \dot{h}{\cdot L}_{CEm}\cdot{Fmax}_{m}+\dot{w}_{m}$ *(17)*

where $\dot{h}$ is the total metabolic cost due to energy losses from heat associated with the simulated muscles' activation, maintenance, and shortening-lengthening.

*Margaria, 1968, muscle-based.* This method was originally based on data relating metabolic cost to whole body work rate during uphill and downhill walking [15]. The equation has also been used to effectively estimate steady-state metabolic cost from muscle fibre work rate [10]. We used the following equation:

$Y_{Margaria-muscles}= \sum_{m=1}^{muscle 9} \frac{\dot{w}_{m}}{h}$ *(18)*

where $h$ represents the efficiency which is set at +25% for shortening contractions and -120% for lengthening contractions.

*Umberger et al, 2003.* This model used the activation, fibre force, fibre length, and fibre contraction velocity of the simulated muscles of the right leg as inputs [11]:

$Y_{Umberger}= \sum_{m=1}^{muscle 9} \dot{h}{AM}_{m}+ \dot{h}{SL}_{m}+ \dot{w}_{m}$ *(19)*

where $\dot{h}AM$ is the metabolic cost due to energy losses from heat associated with both activation and maintenance, and $\dot{h}{SL}_{m}$ is the metabolic costs due to shortening and lengthening heat rate.

Model-based metabolic costs used in the human experiment dataset

*Beck et al., 2019.* We generated this within-stride metabolic cost in the human experiment dataset using the sum of muscle activations multiplied by muscle masses as described by Beck et al., (2019) [26]. This type of approach has been used in studies such as Jackson and Collins, (2015) [18]:

$Y_{Beck}=\left( \sum_{m=muscle1}^{muscle7} {A^{'}}_{m} · m_{m} \right)· constant$ *(20)*

where ${A'}_{m}$ is the muscle activation estimated from surface EMG measurements and $m_{m}$ is the estimated muscle mass. As outlined in Beck et al., we do not know the scaling constant needed to match the units of metabolic cost (W kg^-1^). However, we only evaluate the correlation between $Y_{Beck}$ and our perturbation-based method's estimate of $Y_{Beck}$, hence the value of this $constant$ does not matter. We set the constant such that the peak value of the unperturbed condition was 100%.

*Kim and Roberts, 2015.* We generated this within-stride metabolic cost that uses only joint kinematics and kinetics [9]:

$Y_{Kim}= \sum_{j=1}^{joint3} (\tau_{j}w_{j}) +\sum_{j=1}^{joint3} (\dot{h}{AM}_{j}\left| \tau_{j} \right| + \dot{h}{SL}_{j}\left| \tau_{j}w_{j} \right|+ \dot{h}{CC}_{j} )$ *(21)*

where $\tau_{j}$ is the joint moment, $w_{j}$ is the joint angular velocity, $\dot{h}{AM}_{j}$ is an estimate of activation-maintenance heat rate based on joint angular velocity and joint moment, $\dot{h}{SL}_{j}$ is an estimate of shortening-lengthening heat rate based on joint power and a direction-dependent constant, and $\dot{h}{CC}_{j}$ is contraction heat rate constant. We did not need $\dot{h}{CC}_{j}$ since it is a constant and therefore did not matter for evaluating the Pearson correlation between estimated and actual within-stride metabolic cost.

*Margaria 1968, COM-based.* For this method, we applied the relationship between work and metabolic cost established by Margaria to estimate metabolic cost based on centre of mass (COM) work rate of the right leg ($\dot{w}_{COM})$ [27] as in other studies [20]:

$Y_{Margaria-COM}= \frac{\dot{w}_{COM}}{\eta'}$ *(22)*

where $h'$ represents the efficiency which is set at +25% for positive mechanical work and -120% for negative mechanical work.

*Margaria 1986, joint-based.* We applied the relationship between work and metabolic cost established by Margaria to estimate the metabolic cost using the work rate from the ankle, knee, and hip joint of the right leg as in other studies [20]:

$Y_{Margaria-joint}= \sum_{j=1}^{joint 3} \frac{\dot{w}_{j}}{h'}$  *(23)*

*Minetti and Alexander, 1997.* We estimated metabolic cost based on joint moments and joint angular velocity using equations from Minetti and Alexander as implemented in Faraji et al. and Srinivasan [16,28,29].

$Y_{Minetti}= \sum_{j=1}^{joint3} |\tau_{j}| \omega_{max}\Phi(\omega/\omega_{max})$ *(24)*

$\Phi= \frac{0.054+0.056(\omega/\omega_{max})+{(\omega/\omega_{max})}^{2}}{1-1.13(\omega/\omega_{max})+12.8{(\omega/\omega_{max})}^{2}-1.64{(\omega/\omega_{max})}^{2}}$ *(25)*

where $\omega_{max}$ is the maximum angular velocity for the specific joint and the current direction of the joint. We assumed maximum angular velocities of 12, 18, 16, 24, 13.2, and 13.2 rad s^-1^ for plantarflexion, dorsiflexion, knee extension, knee flexion, hip flexion, and hip extension based on [28,30]. $\Phi(\omega/\omega_{max})$ is an angular-velocity-dependent cost coefficient [16].

**References**

1. Song S, Geyer H. 2015 A neural circuitry that emphasizes spinal feedback generates diverse behaviours of human locomotion. *J. Physiol.* **593**, 3493–3511. (doi:10.1113/JP270228)

2. Song S, Geyer H. 2018 Predictive neuromechanical simulations indicate why walking performance declines with ageing. *J. Physiol.* **596**, 1199–1210. (doi:10.1113/JP275166)

3. Antonellis P, Mohammadzadeh Gonabadi A, Myers SA, Pipinos II, Malcolm P. 2022 Metabolically efficient walking assistance using optimized timed forces at the waist. *Sci. Robot.* **7**. (doi:10.1126/scirobotics.abh1925)

4. Crowninshield RD, Brand RA. 1981 A physiologically based criterion of muscle force prediction in locomotion. *J. Biomech.* **14**, 793–801. (doi:10.1016/0021-9290(81)90035-X)

5. Thelen DG, Anderson FC. 2006 Using computed muscle control to generate forward dynamic simulations of human walking from experimental data. *J. Biomech.* **39**, 1107–1115. (doi:10.1016/j.jbiomech.2005.02.010)

6. Hansen N. 2006 Towards a new evolutionary computation. *Stud. Fuzziness Soft Comput.* **192**, 75–102.

7. Gonabadi AM, Antonellis P, Malcolm P. 2020 A System for Simple Robotic Walking Assistance With Linear Impulses at the Center of Mass. *IEEE Trans. Neural Syst. Rehabil. Eng.* **28**, 1353–1362. (doi:10.1109/TNSRE.2020.2988619)

8. Antonellis P, Frederick CM, Gonabadi AM, Malcolm P. 2020 Modular footwear that partially offsets downhill or uphill grades minimizes the metabolic cost of human walking. *R. Soc. Open Sci.* **7**, 191527–191527. (doi:10.1098/rsos.191527)

9. Kim JH, Roberts D. 2015 A joint-space numerical model of metabolic energy expenditure for human multibody dynamic system. *Int. J. Numer. Methods Biomed. Eng.* **31**, e02721–e02721. (doi:10.1002/cnm.2721)

10. Koelewijn AD, Heinrich D, van den Bogert AJ. 2019 Metabolic cost calculations of gait using musculoskeletal energy models, a comparison study. *PLOS ONE* **14**, e0222037–e0222037. (doi:10.1371/journal.pone.0222037)

11. Umberger BR, Gerritsen KGM, Martin PE. 2003 A Model of Human Muscle Energy Expenditure. *Comput. Methods Biomech. Biomed. Engin.* **6**, 99–111. (doi:10.1080/1025584031000091678)

12. Houdijk H, Bobbert MF, de Haan A. 2006 Evaluation of a Hill based muscle model for the energy cost and efficiency of muscular contraction. *J. Biomech.* **39**, 536–543. (doi:10.1016/j.jbiomech.2004.11.033)

13. Bhargava LJ, Pandy MG, Anderson FC. 2004 A phenomenological model for estimating metabolic energy consumption in muscle contraction. *J. Biomech.* **37**, 81–88. (doi:10.1016/S0021-9290(03)00239-2)

14. Lichtwark GA, Wilson AM. 2005 Effects of series elasticity and activation conditions on muscle power output and efficiency. *J. Exp. Biol.* **208**, 2845–2853. (doi:10.1242/jeb.01710)

15. Margaria R. 1968 Positive and negative work performances and their efficiencies in human locomotion. *Int. Z. Angew. Physiol. Einschlieulich Arbeitsphysiologie* **25**, 339–351. (doi:10.1007/BF00699624)

16. Minetti AE, Alexander RMcN. 1997 A Theory of Metabolic Costs for Bipedal Gaits. *J. Theor. Biol.* **186**, 467–476. (doi:10.1006/jtbi.1997.0407)

17. Beck ON, Shepherd MK, Rastogi R, Martino G, Ting LH, Sawicki GS. 2023 Exoskeletons need to react faster than physiological responses to improve standing balance. *Sci. Robot.* **8**. (doi:10.1126/scirobotics.adf1080)

18. Jackson RW, Collins SH. 2015 An experimental comparison of the relative benefits of work and torque assistance in ankle exoskeletons. *J. Appl. Physiol.* **119**, 541–557. (doi:10.1152/japplphysiol.01133.2014)

19. Miller RH, Brandon SCE, Deluzio KJ. 2013 Predicting Sagittal Plane Biomechanics That Minimize the Axial Knee Joint Contact Force During Walking. *J. Biomech. Eng.* **135**. (doi:10.1115/1.4023151)

20. Caputo JM, Collins SH. 2015 Prosthetic ankle push-off work reduces metabolic rate but not collision work in non-amputee walking. *Sci. Rep.* **4**, 7213–7213. (doi:10.1038/srep07213)

21. Roberts D, Hillstrom H, Kim JH. 2016 Instantaneous Metabolic Cost of Walking: Joint-Space Dynamic Model with Subject-Specific Heat Rate. *PLOS ONE* **11**, e0168070–e0168070. (doi:10.1371/journal.pone.0168070)

22. Jackson RW, Dembia CL, Delp SL, Collins SH. 2017 Muscle-tendon mechanics explain unexpected effects of exoskeleton assistance on metabolic rate during walking. *J. Exp. Biol.* (doi:10.1242/jeb.150011)

23. Mohammadzadeh Gonabadi A, Antonellis P, Malcolm P. 2020 Differences between joint-space and musculoskeletal estimations of metabolic rate time profiles. *PLOS Comput. Biol.* **16**, e1008280–e1008280. (doi:10.1371/journal.pcbi.1008280)

24. Markowitz J, Herr H. 2016 Human Leg Model Predicts Muscle Forces, States, and Energetics during Walking. *PLOS Comput. Biol.* **12**, e1004912–e1004912. (doi:10.1371/journal.pcbi.1004912)

25. Pimentel RE, Pieper NL, Clark WH, Franz JR. 2021 Muscle metabolic energy costs while modifying propulsive force generation during walking. *Comput. Methods Biomech. Biomed. Engin.* **24**, 1552–1565. (doi:10.1080/10255842.2021.1900134)

26. Beck ON, Punith LK, Nuckols RW, Sawicki GS. 2019 Exoskeletons Improve Locomotion Economy by Reducing Active Muscle Volume. *Exerc. Sport Sci. Rev.* **47**, 237–245. (doi:10.1249/JES.0000000000000204)

27. Kuo AD, Donelan JM, Ruina A. 2005 Energetic consequences of walking like an inverted pendulum: Step-to-step transitions. *Exerc. Sport Sci. Rev.* (doi:10.1097/00003677-200504000-00006)

28. Faraji S, Wu AR, Ijspeert AJ. 2018 A simple model of mechanical effects to estimate metabolic cost of human walking. *Sci. Rep.* **8**, 10998–10998. (doi:10.1038/s41598-018-29429-z)

29. Srinivasan M. 2011 Fifteen observations on the structure of energy-minimizing gaits in many simple biped models. *J. R. Soc. Interface* **8**, 74–98. (doi:10.1098/rsif.2009.0544)

30. Geyer H, Herr H. 2010 A Muscle-Reflex Model That Encodes Principles of Legged Mechanics Produces Human Walking Dynamics and Muscle Activities. *IEEE Trans. Neural Syst. Rehabil. Eng.* **18**, 263–273. (doi:10.1109/TNSRE.2010.2047592)

Data S1. Code for perturbation-based method. This ZIP file contains a version of the neuromechanical simulation dataset and the human experiment dataset, each with five different model-based versions of metabolic cost, a MATLAB script that uses the perturbation-based method to estimate each of the model-based within-stride costs, and a README file with documentation. This ZIP file also contains the human experimental stride-mean metabolic cost data from respiratory V̇O_2_ and V̇CO_2._

**Data S2. Code for tuning of the perturbation-based method.** This ZIP file contains a version of the neuromechanical simulation dataset with 100 randomly generated time series that are used as estimation targets for the tuning, a MATLAB that calculates the optimal settings for the selection of mathematically derived time series to consider and the bin size, and a README file with documentation.
